# Insights into the context-dependent immunological roles of the CXCL12–CXCR4 axis in alopecia

**DOI:** 10.3389/fimmu.2026.1753817

**Published:** 2026-04-15

**Authors:** Jong-Hyuk Sung, Mei Zheng, In Guk Park, Seungchan An, Jayhyun Cho, Minsoo Noh

**Affiliations:** 1 Epi Biotech Co., Ltd., Incheon, Republic of Korea; 2 College of Pharmacy and Natural Products Research Institute, Seoul National University, Seoul, Republic of Korea

**Keywords:** alopecia, CXCL12-CXCR4 axis, hair-loss therapeutics, macrophages, regulatory T cells

## Abstract

The chemokine CXCL12 and its receptor CXCR4 play context-dependent roles in hair follicle biology. While recent findings suggest that regulatory T cells (Tregs) utilize the CXCL12–CXCR4 axis to modestly promote hair follicle regeneration under homeostatic conditions, a substantial body of evidence indicates that this same axis principally drives pathological processes leading to hair loss in alopecia. In androgenetic alopecia (AGA) and alopecia areata (AA) – the most common forms of hair loss – CXCL12–CXCR4 signaling fosters a fibroimmune microenvironment characterized by dermal fibrosis, chronic inflammation, and hair follicle miniaturization. CXCR4 expression in diseased scalp is found predominantly on pro-inflammatory macrophages and dermal papilla cells (DPCs), rather than on Tregs, implicating these cells in propagating hair follicle damage. Correspondingly, elevated CXCL12 from dermal fibroblasts recruits immune effectors and enhances CXCR4 signaling in follicular cells, linking hormonal or autoimmune triggers to hair follicle destruction. Treg-expressed CXCR4 contributes only a minor, context-dependent influence on hair growth, often overwhelmed by the potent pathological signals in alopecia. Therapeutically, inhibiting the CXCL12–CXCR4 axis in both AGA and AA models consistently reverses fibrosis, curtails pathogenic immune cell accumulation, restores DPC function, and stimulates robust hair regrowth. This perspective synthesizes current evidence on: (1) the cellular sources of CXCR4 in alopecic tissue; (2) the pathogenic role of CXCL12–CXCR4 signaling in AGA and AA; (3) the limited scope of Treg CXCR4 function in healthy hair growth; (4) outcomes of CXCL12/CXCR4 blockade as a treatment strategy; and (5) key confounding factors to consider when interpreting CXCR4’s role in hair biology.

## 1 Introduction

Alopecia, including androgenetic alopecia (AGA) and alopecia areata (AA), involves complex interactions between the immune system, hair follicle niche cells, and signaling pathways. The CXCL12–CXCR4 chemokine axis has emerged as a critical player in these interactions. A recent study by *Cohen* et al. (2025) reported that Tregs in skin utilize CXCR4 to home to hair follicles, resulting in modestly enhanced hair regeneration (1). Similarly, Zhao et al. (2024) found that CXCL12 (also known as SDF-1) promotes hair follicle growth in a chronic stress-induced hair loss model, an effect reversed by the CXCR4 antagonist AMD3100 (2). These findings highlight specific homeostatic or stress-related contexts in which CXCL12–CXCR4 signaling can support hair growth. However, our studies in disease models of alopecia support an alternative model wherein CXCL12/CXCR4 drives a pathogenic fibroimmune microenvironment that leads to hair follicle degeneration and hair loss (3–7). In other words, the same chemokine axis that provides minor pro-growth cues in healthy conditions predominantly mediates detrimental fibrosis and inflammation in alopecia.

Functional divergence of the CXCL12–CXCR4 axis is governed by upstream microenvironmental reprogramming rather than intrinsic duality of the pathway. Under homeostatic conditions, low-level keratinocyte-derived CXCL12 establishes localized gradients that preferentially support CXCR4^+^ Treg positioning within the follicular niche, facilitating immune-regulated regeneration. In contrast, during alopecia, androgen signaling (AGA) or inflammatory cytokines such as IFN-γ (AA) markedly upregulate CXCL12 in dermal fibroblasts, shifting the principal chemokine source from epithelial to mesenchymal compartments (1, 3, 4). This shift is accompanied by expansion of CXCR4-high dermal papilla cells and infiltrating pro-inflammatory macrophages, while Tregs constitute a minor fraction of CXCR4+ cells in diseased tissue. Elevated chemokine gradients, altered receptor density, immune privilege collapse, and fibroinflammatory remodeling collectively redirect CXCL12 responsiveness toward pathogenic myeloid and mesenchymal populations (4). Thus, tissue context, cellular composition, chemokine gradients, and receptor expression levels determine the dominant biological output of CXCL12–CXCR4 signaling.

In AGA, CXCR4 activation in DP cells induces functional reprogramming characterized by enhanced androgen receptor signaling, reduced expression of pro-regenerative growth factors, and increased expression of matrix-remodeling enzymes such as MMPs (4, 5, 7). Single-cell transcriptomic analyses support a model of DP transcriptional reprogramming rather than simple numerical depletion, linking CXCL12–CXCR4 signaling to impaired hair matrix cell support, premature catagen entry, and progressive follicular miniaturization (4). Together, macrophage-mediated immune amplification and DP-centered mesenchymal dysfunction provide a unified downstream mechanism connecting CXCL12–CXCR4 activation to hair cycle disruption and translational therapeutic targeting. In AA, CXCL12-driven recruitment and retention of pro-inflammatory dendritic cells or macrophages amplifies IFN-γ–dependent signaling, promotes collapse of hair follicle immune privilege, and enhances cytotoxic CD8+ T-cell infiltration (3). This inflammatory amplification sustains a feed-forward loop of cytokine production and tissue damage.

Therefore, this perspective examines the evidence that CXCL12–CXCR4 predominantly acts as a pathogenic driver in alopecia, overshadowing any limited regenerative role. We focus on four key aspects of this paradigm:

- CXCR4 expression in alopecic tissue is concentrated in pathogenic macrophages and dermal papilla cells (DPCs), not Tregs.
- In both AGA and AA, CXCL12–CXCR4 signaling orchestrates pathological “fibroimmune” remodeling and follicle miniaturization.
- Treg-expressed CXCR4 plays a modest, context-dependent role in hair maintenance, with minimal influence during disease.
- Therapeutic blockade of the CXCL12–CXCR4 axis in alopecia models reverses pathology and induces hair regrowth.

In the sections below, we discuss each of these points in detail, drawing on evidence from both murine models and clinical observations to clarify the role of CXCL12–CXCR4 in hair loss disorders.

## 2 CXCR4 expression in alopecic tissue: macrophages and DPCs versus Tregs

Studies of alopecia indicate that CXCR4 is chiefly expressed on pro-inflammatory macrophages and on hair follicle dermal papilla cells (DPCs) in diseased scalp, rather than on Tregs (3, 4, 7). In androgenetic alopecia, for example, CXCR4 is significantly upregulated in the dermal papilla and outer root sheath of balding hair follicles (7). Its expression is further elevated in DPCs under alopecic (androgen-exposed) conditions, suggesting that follicular cells themselves upregulate CXCR4 during disease. Dermal fibroblast–derived CXCL12 appears to drive this upregulation: adding exogenous CXCL12 increases CXCR4 levels in adjacent DPCs, creating an autocrine/paracrine loop that amplifies androgen receptor (AR) signaling in the follicle and thereby promotes follicle miniaturization (5).

Similarly, single-cell transcriptomic analyses of androgenic areata (AGA) lesions show high Cxcr4 expression on infiltrating myeloid cells – notably TREM2^+^ pro-fibrotic macrophages – and on hair follicle-associated mesenchymal cells, whereas Foxp3^+^ Tregs comprise only a small fraction of the CXCR4^+^ cell population (4). In an alopecia areata (AA) mouse model (induced by pathogenic lymphocyte transfer), the vast majority of CXCR4-expressing cells accumulating around hair follicles were found to be pathogenic immune cells (e.g. cytotoxic CD8^+^ T cells, dendritic cells, and macrophages) rather than Tregs (3, 4). These observations underscore that in alopecic conditions, CXCL12/CXCR4 signaling predominantly engages macrophages and follicular stromal cells that propagate hair follicle damage, instead of acting through Tregs.

**Figure 1:**
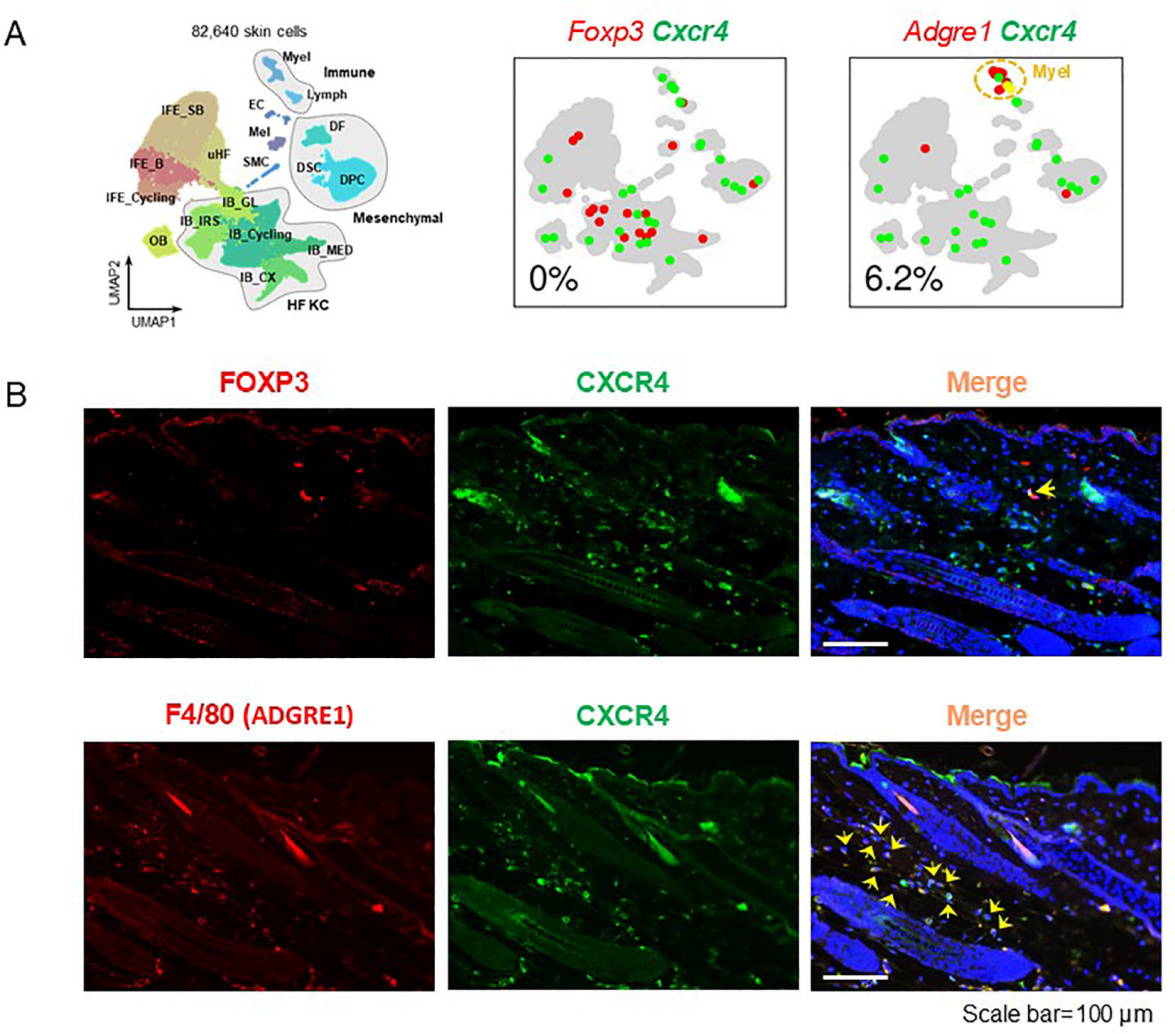
CXCR4 expression is predominantly associated with macrophages (ADGRE1^+^ cells) rather than Tregs in skin. **(A)** Single-cell RNA sequencing analysis (GSE295410) of mouse back skin shows that *Cxcr4* mRNA is co-expressed with *Adgre1* (encoding F4/80, a macrophage marker) but not with *Foxp3* (a Treg marker). **(B)** Immunofluorescence staining of mouse skin demonstrates that CXCR4 protein (green) co-localizes with ADGRE1^+^ macrophages (red) and not with FOXP3^+^ Tregs. These data confirm that CXCR4 is primarily expressed by macrophages in mouse skin. Scale bar=100 μm.

Consistent with these findings, our analysis of murine skin confirms that CXCR4 is associated with macrophages, not Tregs. Single-cell RNA sequencing data from mouse back skin reveals that Cxcr4 transcripts are co-expressed with Adgre1 (encoding F4/80, a macrophage marker) and not with Foxp3 (a Treg marker) (**Figure 1a**). Likewise, immunofluorescence imaging shows CXCR4 protein colocalizing with F4/80^+^ macrophages but not with FOXP3^+^ Tregs in skin (**Figure 1b**). These results (summarized in **Figures 1a, b**) indicate that CXCR4 is primarily expressed by macrophages in alopecic tissue, with Tregs representing a minor CXCR4^+^ subset. Indeed, even in the absence of disease, disrupting CXCL12–CXCR4 signaling produces only a “suboptimal” (slightly reduced) hair growth phenotype (1). This modest effect size aligns with the idea that Tregs are secondary players in this pathway *in vivo*. In summary, the cellular sources and targets of CXCL12/CXCR4 in hair-loss conditions are fundamentally different from those in homeostatic Treg trafficking – pathogenic stromal and myeloid cells dominate CXCR4-dependent processes in alopecia.

## 3 Pathogenic CXCL12–CXCR4 signaling in AGA and AA

**Figure 2:**
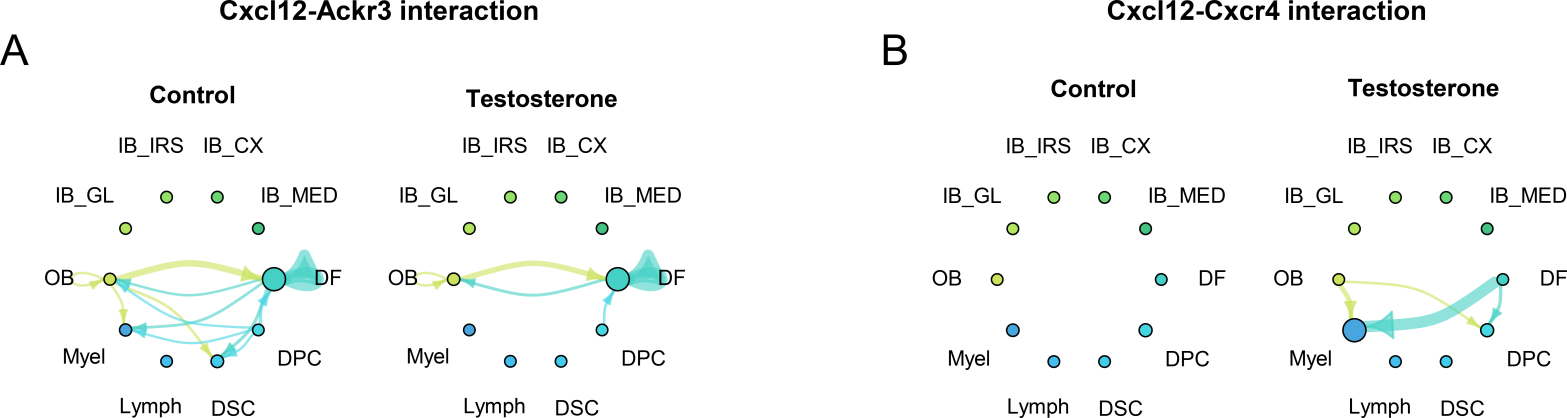
CellChat analysis of Cxcl12–Ackr3 and Cxcl12–Cxcr4 interactions in testosterone propionate–treated mouse dorsal skin (GSE295410). **(A)** Network visualization of Cxcl12–Ackr3 signaling inferred from single-cell RNA-seq data, showing directional communication probability between major skin cell populations after androgen stimulation. Line thickness represents interaction strength. **(B)** Cxcl12–Cxcr4 signaling network under the same condition, highlighting distinct receptor-specific communication patterns between dermal fibroblast (DF) and Myeloid cells (Myel) after androgen stimulation.

Compelling evidence links the CXCL12–CXCR4 axis to the core pathological changes in both AGA and AA (3, 4). In AGA – the androgen-driven form of pattern hair loss –CXCL12 serves as a critical bridge between androgen signaling and downstream fibroimmune tissue remodeling (**Figure 2**). Dermal fibroblasts in balding scalp are highly androgen-responsive and are the primary source of excess CXCL12 under androgen exposure (5). This fibroblast-derived CXCL12 has dual pathogenic actions: (1) autocrine signaling via CXCL12’s alternative receptor CXCR7 (ACKR3) on those fibroblasts activates TGF-β pathways, driving dermal fibrosis (excess extracellular matrix deposition); (2) paracrine signaling via CXCR4 reprograms neighboring DPCs and recruits myeloid cells such as macrophages to the follicle niche (3, 4). Notably, CXCL12/CXCR4 activity in AGA promotes the accumulation of TREM2^+^ macrophages – a population of profibrotic, inflammation-associated macrophages – around hair follicles. The net result is “fibroimmune” remodeling of the tissue, characterized by concurrent dermal fibrosis, chronic inflammation, and hair follicle miniaturization leading to hair loss. Single-cell RNA sequencing of an AGA mouse model further illuminated this mechanism: testosterone treatment upregulated Cxcl12 (~2.8-fold) and Cxcr4 (~6.2-fold) in scalp tissue, and network analysis identified CXCL12 signaling as central to the observed stromal and immune dysregulation (3, 4). Crucially, pharmacologically blocking CXCL12 in this model prevented the testosterone-induced fibrotic and inflammatory changes, demonstrating that CXCL12–CXCR4 is a necessary mediator of androgen-driven pathological remodeling in AGA.

In AA, an autoimmune form of hair loss, a similar pathological CXCL12–CXCR4 axis operates despite the different disease trigger (3). AA lesions show markedly elevated CXCL12 production by dermal fibroblasts, especially under inflammatory cytokine cues like interferon-γ. The aberrant CXCL12 creates an “inflammatory milieu” that attracts and activates immune effectors against the hair follicle (3). Indeed, CXCL12 can directly stimulate T cells (both CD4^+^ helper and CD8^+^ cytotoxic subsets) that drive AA progression. Furthermore, recent studies have uncovered a role for innate γδ T cells as early responders in AA (8). Vδ1^+^ γδ T cells, which are sparse and quiescent in healthy scalp, were found to infiltrate the peribulbar region of AA hair follicles – even in clinically unaffected (“non-lesional”) areas – and to exhibit a pro-inflammatory, IFN-γ–producing phenotype. Notably, lesional AA follicles showed elevated CXCL12 expression, a chemoattractant that likely recruits and activates these γδ T cells (9). Mechanistic experiments confirm that human dermal γδ T cells can directly recognize “stressed” hair follicles and trigger immune privilege collapse and premature catagen (hallmark features of AA) via CXCL12-mediated activation. Thus, CXCL12 not only attracts conventional αβ T cells but can also engage innate γδ T cells that serve as stress sentinels to initiate hair follicle autoimmunity (9). Fibroblast-derived CXCL12 has been shown to induce the collapse of the hair follicle’s immune privilege, thereby allowing autoreactive CD8^+^ T cells to infiltrate the follicle and attack it (3). In AA mouse models, an excess of CXCL12 correlates with increased infiltration of CD8^+^ T cells and upregulation of MHC class I/II in the skin, whereas neutralizing CXCL12 significantly reduces these pathogenic infiltrates. Importantly, AA mice treated with CXCL12-blocking agents show preservation of hair follicles and a delayed onset of disease (3), underscoring that CXCL12–CXCR4 signaling reinforces the self-perpetuating loop of immune attack and tissue remodeling in AA.

Thus, in both AGA and AA, CXCL12 is not merely a benign chemotactic signal; rather, it is a driver of destructive fibroinflammatory changes and follicular degeneration that culminate in hair loss (7). The pathogenic actions of this axis link the initiating factors of each disease (androgens in AGA, autoimmunity in AA) to a common outcome: a hostile microenvironment that undermines hair follicle integrity. Targeting this pathway therefore holds promise for interrupting disease progression in alopecia (discussed further in Section 4).

## 4 Treg CXCR4 signaling in homeostasis: context-dependent, limited influence

Regulatory T cells are important for skin immune homeostasis and have been implicated in facilitating hair follicle regeneration in certain contexts (10, 11). However, the contribution of Treg-expressed CXCR4 to hair growth appears limited in scope and highly context-dependent. Cohen et al. demonstrated that Tregs use CXCR4 to localize to hair follicles and that Treg-specific loss of CXCR4 leads to mildly impaired (“suboptimal”) hair regrowth after hair plucking (1). We interpret this to mean that under non-diseased conditions, CXCR4 helps Tregs optimally position themselves in the skin to support regeneration, but the magnitude of this effect is modest. Moreover, hair follicle development itself appears not to require CXCR4. Cxcr4 is transiently expressed in embryonic hair follicle placodes and dermal condensates, but mice with Cxcr4 deficiency still form normal hair follicles (12). This suggests that CXCL12/CXCR4 signaling is dispensable for the fundamental morphogenesis of hair follicles, aligning with its relatively minor role in baseline hair growth. Even this modest pro-growth influence may depend on specific physiological conditions. Notably, Cohen et al. reported that Treg Cxcr4 expression is partly dependent on glucocorticoid receptor (GR) signaling, suggesting that stress hormones or exogenous steroids can modulate Treg homing via CXCR4. Glucocorticoids themselves are known to affect hair cycling (often promoting catagen, the regression phase, via factors like DKK-1), which means that elevated glucocorticoid signaling in an experimental system could confound the interpretation of Treg-specific effects on hair growth (13, 14). In other words, some of the hair phenotype seen upon Treg CXCR4 deletion might result from glucocorticoid-induced hair cycle changes rather than the absence of Treg trafficking alone.

Crucially, in disease contexts such as AGA or AA, any hair-promoting influence of Treg CXCR4 is overwhelmed by pathological factors. In AA, for instance, Tregs are present in lesions (and may even be somewhat elevated in number), yet they fail to prevent disease progression amid the onslaught of autoreactive CD8^+^ T cells attacking the follicles (15, 16). Single-cell analyses of human AA lesions have shown increases in Treg populations alongside other T cell subsets, but the dominant drivers of hair follicle destruction are the cytotoxic CD8^+^ T cells – and Tregs, even if present, cannot fully counterbalance their activity. This is consistent with functional studies where depleting Tregs in AA has only a minimal effect on disease severity compared to the effect of removing effector T cells. Likewise in AGA, there is little evidence for a significant protective or regenerative role of Tregs; AGA pathology is defined by low-grade chronic inflammation and fibrosis, not by a failure of Treg-mediated support. In such conditions, boosting Treg numbers or function (with or without CXCR4) is insufficient to overcome the dominant CXCL12/CXCR4-driven damage wrought by dermal fibroblasts, macrophages, and effector T cells.

Therefore, Treg-associated CXCR4 signaling can be seen as a fine-tuning mechanism for hair growth under healthy conditions, but one that is easily eclipsed in the face of pathological alopecia. Any therapeutic strategy aimed at enhancing hair growth by boosting Treg recruitment (e.g. via CXCR4) would need to contend with the much stronger opposing signals of the CXCL12–CXCR4 axis in diseased tissue. In practice, the data suggest that targeting the root pathogenic process (fibrosis/immune recruitment via CXCL12–CXCR4) is far more impactful for hair restoration than trying to amplify Tregs’ minor homeostatic role (4).

## 5 Therapeutic inhibition of the CXCL12–CXCR4 axis in alopecia

Perhaps the most compelling evidence for the pathogenic role of CXCL12–CXCR4 in alopecia comes from interventions that block this axis, which have shown striking success in rescuing hair growth. Multiple independent studies in both AGA and AA models have demonstrated that neutralizing CXCL12 or pharmacologically inhibiting CXCR4 reverses key pathological features and promotes robust hair regeneration (3, 4, 6).

In a controlled *in vitro* organ culture model of hair follicles, the addition of recombinant CXCL12 was found to inhibit hair growth (delaying entry into the anagen growth phase and resulting in shorter hair shafts). Conversely, applying a CXCR4 antagonist or CXCL12-neutralizing antibody accelerated anagen initiation and led to significantly increased hair growth in these cultures. These findings establish a direct causal link between CXCL12–CXCR4 signaling and hair follicle growth dynamics: CXCL12 signaling is sufficient to impair hair growth, and its inhibition actively promotes hair shaft elongation.

*In vivo* studies, therapeutic blockade of CXCL12–CXCR4 has yielded dramatic improvements in alopecia models. For example, Zheng et al. treated AGA-model mice (which were undergoing testosterone-induced hair follicle miniaturization) with a CXCL12-neutralizing antibody (4). Despite continued androgen exposure, the treated mice showed marked increases in hair density and hair follicle size relative to controls (6). The anti-CXCL12 therapy effectively protected the follicles from androgen-mediated damage and rescued them from the progressive miniaturization seen in untreated AGA mice. Mechanistic analysis revealed that blocking CXCL12 normalized the molecular and cellular environment of the scalp: it reduced elevated AR and CXCR4 expression in DPCs (disrupting the pathological positive feedback loop), and it prevented the expansion of pro-fibrotic dermal fibroblast populations and infiltrating macrophages in the skin. As a result, the dermal papilla regained its regenerative signaling capacity and previously thinning follicles began to produce thicker, normal-caliber hairs. These outcomes highlight the therapeutic potential of targeting CXCL12/CXCR4 in AGA, effectively counteracting the androgen-driven pathogenic mechanism.

In autoimmune alopecia (AA) models, inhibition of the CXCL12–CXCR4 axis has shown equally promising results. AA-prone mice treated with a humanized anti-CXCL12 antibody exhibited significantly delayed onset of hair loss and reduced disease severity compared to untreated controls (3). Histological examination of treated AA mice revealed a marked decrease in the accumulation of inflammatory cells in the skin: the dense infiltrates of T cells and dendritic cells/macrophages typical of AA lesions were substantially diminished by CXCL12 blockade. Gene expression analyses further showed that key inflammatory chemokines and cytokines (e.g. Ifng, Ccl5, Ccr5) which are highly upregulated in active AA lesions were downregulated upon CXCL12 inhibition, consistent with an overall dampening of the pathogenic immune response. Notably, blocking CXCR4 signaling reduced the aberrant retention of CD8^+^ cytotoxic T cells within hair follicles – these cells are central mediators of follicle destruction in AA – thereby protecting the follicles from immune attack. Treated mice maintained intact, cycling hair follicles and showed ongoing hair growth, whereas control AA mice displayed follicle dystrophy and extensive hair loss.

Collectively, these findings illustrate that therapeutic interruption of CXCL12–CXCR4 signaling can reset the alopecic scalp/skin microenvironment from a hair-loss state toward a hair-favorable state. By halting the CXCL12-driven fibrotic and inflammatory cascade, such treatments alleviate the key pathological drivers: dermal fibrosis is reduced, pro-inflammatory macrophages either exit or revert to a repair phenotype, DPCs resume normal hair-supportive signaling, and autoreactive T cells are kept at bay. The outcome is significant hair regrowth and restoration of healthy follicles.

## 6 Conclusion

In summary, current evidence strongly supports a model of alopecia in which dermal fibroblast–derived CXCL12 recruits macrophages and stimulates CXCR4^+^ DPCs, creating a pro-fibrotic, immune-dysregulated niche that drives hair follicle destruction. In this disease context, CXCR4 emerges as a facilitator of follicle miniaturization and hair loss, in stark contrast to the limited, auxiliary role it plays in Tregs under healthy conditions. While recent findings on Treg CXCR4 are insightful, they delineate a narrow scenario of homeostatic hair cycle regulation and do not refute the predominantly pathogenic role of CXCL12–CXCR4 in alopecia. The preponderance of data from AGA and AA models shows that blocking CXCL12/CXCR4 signaling leads to significant hair regrowth, reduced fibrosis and inflammation, and normalization of follicular function– precisely the outcomes desired in treating hair loss. It is therefore critical to distinguish the context of homeostatic hair-follicle maintenance (where Treg CXCR4 provides a modest benefit) from the context of active alopecia (where CXCL12–CXCR4 is a central pathogenic driver). This distinction has tangible therapeutic implications: it suggests that targeting the CXCL12/CXCR4 axis should be a priority in combating pathological hair loss, whereas any potential side effect on Treg trafficking is likely a minor and manageable trade-off given Tregs’ comparatively small role during disease. Ongoing and future studies in human patients and scalp tissues will further clarify these dynamics, but the evidence to date overwhelmingly implicates CXCL12–CXCR4 as a culprit in hair follicle pathology. Together, these findings reinforce the rationale for making this chemokine axis a focal point in alopecia research and treatment strategies.

## Data Availability

The original contributions presented in the study are included in the article/**Supplementary Material**. Further inquiries can be directed to the corresponding authors.
