# Supplementary figures and images for: Insights into the context-dependent immunological roles of the CXCL12–CXCR4 axis in alopecia
Source: Front Immunol. 2026 Apr 15;17:1753817. doi: 10.3389/fimmu.2026.1753817 (PMC13124579; doi:10.3389/fimmu.2026.1753817)

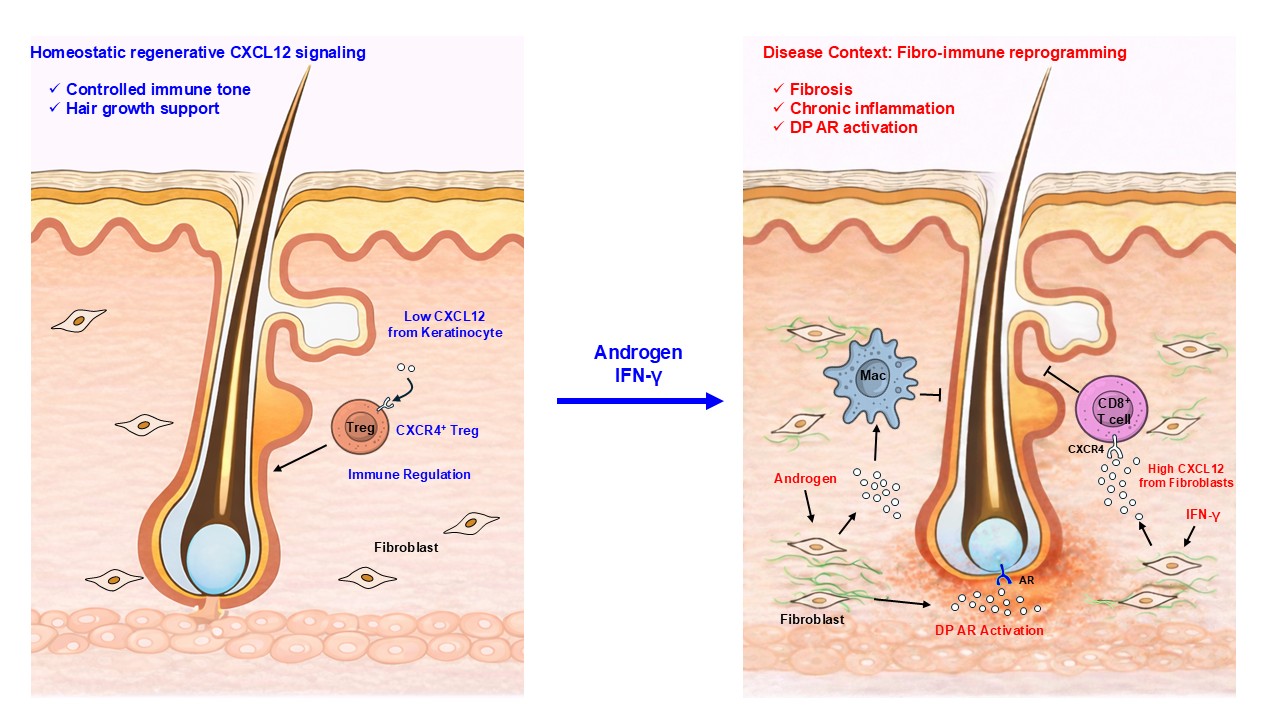

Supplement: Supplementary file 1 [file Image1.jpeg]
